# Soybean replacement value of canola meal as measured by growth performance and feed efficiency in broiler chickens: Insights from a meta-analysis: CANOLA MEAL HAS POOR REPLACEMENT POTENTIAL FOR SOYBEAN IN BROILER DIETS

**DOI:** 10.1016/j.psj.2025.104876

**Published:** 2025-02-20

**Authors:** Freddy Manyeula, Moemedi Dikakanyo Legodimo, John Cassius Moreki, Victor Mlambo

**Affiliations:** a Department of Animal Sciences, Faculty of Animal and Veterinary Sciences, Botswana University of Agriculture and Natural Resources, Gaborone, Botswana; b School of Agricultural Sciences, Faculty of Agriculture and Natural Sciences, University of Mpumalanga, Mbombela, South Africa

**Keywords:** Broiler diets, canola meal, growth performance, moderators, soybean meal

## Abstract

While the use of canola meal (**CM**) as an alternative to soybean meal in broiler diets is well documented, the results are still conflicting. Therefore, this meta-analysis combines results from multiple studies to provide a more precise estimate of the effect size or relationship between dietary CM and feed intake (**FI**), average daily gain (**ADG**), and feed conversion ratio (**FCR**) in broiler chickens. This approach explores the inconsistencies, identifies knowledge gaps, and creates new insights using published data. Search were conducted in Google scholar, Scopus, Web of Sciences, and PubMed, yielding a total of nineteen (19) relevant articles for this study. The data generated analysed using OpenMEE software. Heterogeneity was explored by subgroup and meta-regression analyses using moderator variables (i.e., publication year, strain, gender, inclusion levels, treatment methods, and study periods). The results showed that dietary CM significantly reduced FI [standard mean difference (**SMD**) = −0.33; 95% confidence interval (**CI**) = -0.41 to -0.25] and ADG [SMD = −0.68; 95% CI -0.85 to -0.50] while increasing FCR [SMD = − 0.37; 95% CI = 0.24 to 0.51] compared to the control group. Restricted subset analysis showed that studied moderators influenced the outcomes of this meta-analysis. Meta-regression revealed that the stage of development of the birds and treatment methods on CM were the significant predictors of FI while gender and treatment methods significantly predicted both ADG and FCR. In conclusion, the inclusion of CM in broiler diets resulted in poor growth performances, possibly due to anti-nutritional compounds such as glucosinolates, erucic acid, sinapine, and tannins. Thus, innovative research on processing methods to enhance the soybean replacement value of CM in broiler production is necessary.

## Introduction

Poultry meat is the most consumed source of animal protein globally (Korver, 2023). Its demand is expected to keep on increasing in response to the ever-expanding human population (OECD/FAO, 2024). However, the poultry industry is faced with many challenges such as diseases (Asfaw et al., 2021; Mramba and Nwatambo, 2024) and a lack of good quality alternative protein sources (Janocha et al., 2022). Soybean meal (**SBM**) is the dominant protein source in broiler rations due to its high protein content and superior amino acid profile (Kuenz et al., 2022). However, the use of SBM in broiler diets has been deemed to be economically, environmentally, and socially unsustainable, particularly in drier regions of the world (Disetlhe et al., 2018; Tamasgen et al., 2021). Moreover, several key factors account for the unavailability of SBM in developing countries, including the disruption of imports due to documentation disputes concerning the genetically modified organism (GMO) certifications. Because SBM is primarily imported from nations like the United States and Brazil, which mainly produce GMO variants, delays in receiving regulatory approvals in shipments being held back at ports (Abbas et al., 2023). Accordingly, readily available, less expensive, and high-quality vegetable proteins such as canola meal (CM) are required to replace soybeans in broiler diets. Indeed, in certain regions, agronomic conditions are more favorable for cultivating canola than soybean (Hansen et al., 2020). As a result, incorporating CM into animal diets can enhance the profitability of intensive animal agriculture, contributing to a positive global economic impact. Canola meal is a by-product of canola oil extraction, which has no direct use as food for humans. Its protein content ranges between 36 and 39% while the essential amino acid profile is comparable to that of SBM (Rehman et al., 2018). However, CM contains higher levels of fiber (12%) and antinutritional factors (**ANFs**) such as glucosinolates (3.8 µmol/g) (Disetlhe et al., 2018) that are likely to hinder the growth of broiler chickens by interfering with nutrient digestion and absorption. Despite these drawbacks, canola has lower agronomic requirements compared to soybean. Indeed, the water, fertilizer and pesticides requirements of canola have been reported to be lower than those of soybeans (Zhang et al., 2024). Accordingly, CM remains a pragmatic soybean meal alternative in semi-arid areas with poor soil fertility.

Various studies have reported improved growth performance (Naseem et al., 2006; Manyeula et al., 2020) when CM partially or completely replaced SBM in broiler diets. Conversely, other studies (Min et al., 2011; Gopinger et al., 2014; Yadav et al., 2022) reported reduced feed digestibility and growth performance in broiler chickens fed high inclusion levels of CM (>25 %). They associated these negative outcomes with a high intake of dietary fiber and other ANFs such as glucosinolates, erucic acid, sinapine, and tannins. Accordingly, various approaches to reduce the negative effects of ANFs on CM have been investigated, including fermentation and use of probiotics (Elbaz, 2021), prebiotics (Niu et al., 2023a), humic acids (Disetlhe et al., 2018), and gamma radiation (Gharaghani et al., 2008). Despite efforts to improve the protein value of CM, inconsistent reports on its effect on FI, growth performance, and carcass weights in broiler chickens abound. This may be attributed to many other factors such as seed coat pigments, inclusion levels, bird strains, growth stage of broiler chickens, and effectiveness of treatment methods. These conflicting results hinder the ability to draw reliable conclusions about the SBM replacement value of CM or to develop broadly applicable insights. We believe that this meta-analysis can generate reliable conclusions enabling farmers, researchers, or other stakeholders to make informed decisions on the utility of CM as an alternative protein source in broiler chickens. Currently, there is a significant gap in the literature, with few published meta-analyses assessing the growth performance and feed efficiency outcomes in broilers fed CM. Therefore, this study was designed to resolve the discordance in research findings by considering the effects of bird strains, growth development stages, and CM inclusion levels and treatment methods on FI, feed utilization, and weight gain in broiler chickens. The study analysed the effects of replacing soybean meal with CM in broiler chickens on growth performance and feed efficiency.

## Material and methods

### Literature search and inclusion criteria

**Fig 1:**
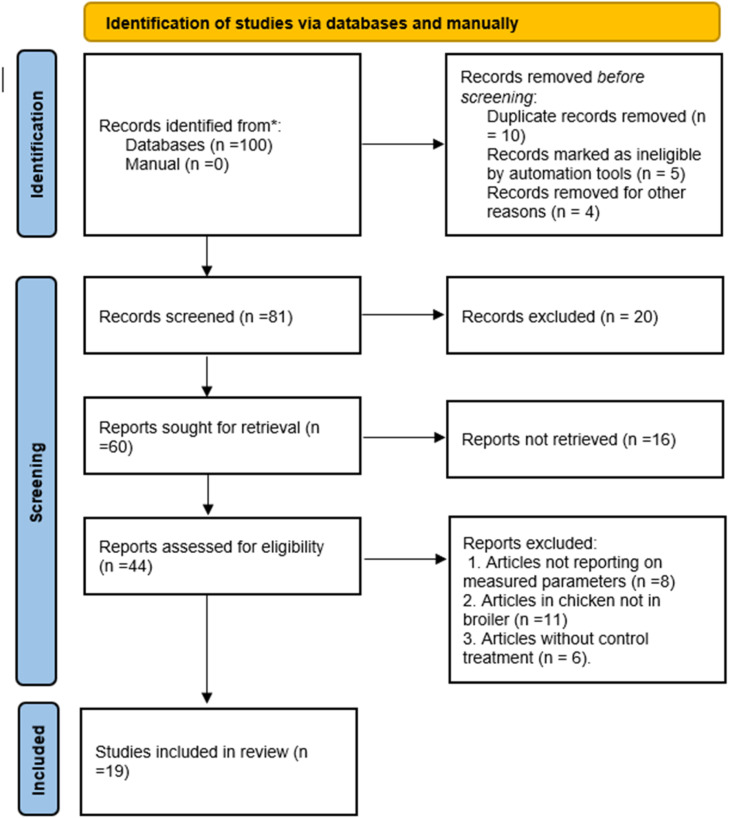
Literature search and selection process following the PRISMA procedure.

Literature search was conducted in Google Scholar (https://scholar.google.com), Scopus (https://www.scopus.com), Web of Sciences (https://www.webofscience.com) and PubMed (https://pubmed.ncbi.nlm.nih.gov) using “growth performance,” “broilers,” “canola meal,” “feeding” and “supplementation**”** search terms, which resulted in a total of 100 papers from 2001 to 2023. After removing duplicate papers, data from eligible articles were captured in an MS Excel file. The titles and abstracts of the articles were examined to exclude studies based on the following criteria: a) papers not focusing on target parameters and broiler chickens; b) grey literature, literature reviews, and unpublished studies; c) papers lacking a control group; and d) papers with treatment groups where conditions, other than the addition of CM to the basal diet, deviated from standard broiler feeding practices; e) a paper without animal ethical clearance. The included articles were studies published in English on broiler chickens, focussing on FI, ADG, and FCR as affected by CM. They provided FI and ADG measurements as either g/bird/day or kg/bird/day. All expressed FI and ADG units were converted to g/bird/day. The full texts of the remaining publications were reviewed, and data on broiler FI, weight gain, and feed efficiency ratio were compiled for potential eligibility classification. A total of nineteen articles deemed to be eligible for inclusion in this meta-analysis, as outlined in the selection process shown in Fig 1.

### Eligibility conditions

The papers were selected using the following inclusion criteria: a) articles that compared FI, ADG, and FCR of broilers; b) articles with detailed descriptions of diet composition; c) articles that reported at least two of these variables: FI, ADG, or FCR; and d) articles that were published in peer-reviewed journals in English. Initially, a total of 100 articles were considered eligible for this meta-analysis and were further screened to 19 articles that met requirement and were used for the analysis as shown by PRISMA flowchart Fig 1 (Moher et al.,2009).

### Data extraction and analysis

Data on the surname of the first author, year of publication, country (USA, Iran, Canada, Brazil, Egypt, Korea, South Africa, and Australia), continents (North and South America, Asia, Africa and Oceania), number of birds used, number of treatments, and moderator variables [strain (Ross, Heritage, Cobb, Leghorn, and Abor Acres), gender (mixed, male and not stated), inclusion levels (0 – 10, 11 – 20, 21 – 30, 31 – 40%) and stage of development (starter, finisher, and overall)] were retrieved from the 19 articles and used for the analysis (Table 1). All the analyses were done in OpenMEE software, which is built in R-software. Effect sizes were pooled using random-effects model (REM) and expressed as standardized mean difference (SMD) at 95% confidence interval (CI). The choice of the REM was based on the assumptions that data included in the meta-analysis were not identical; therefore, variance must be divided into within–studies and between–studies variance plus sampling error (Borenstein et al., 2009).

**Table 1:** Description of articles used in this meta-analysis.

	References	Country	Continent	NBT	NT	Explanatory variables				Outcomes
						Strain	OF	IL	SP	
1	Clark et al. (2001)	USA	NA	390	8	AA	meal	0-30	21	1, 2, 3
2	Gharaghani et al. (2008)	Iran	Asia	80	5	Ross	meal	0-30	42	1, 2, 3
3	Thacker and Petri (2009)	Canada	NA	210	7	Ross	meal	0-15	21	1, 2, 3
4	Woyengo et al. (2011)	Canada	NA	200	5	Ross	meal	0-40	21	1, 2, 3
5	Jung et al. (2012)	USA	NA	920	6	Heritage	meal	0-7.5	49	1, 2, 3
6	Thacker and Widyaratne (2012)	Canada	NA	210	4	Ross	meal	0-15	21	1, 2, 3
7	Gopinger et al. (2014)	Brazil	SA	320	5	Cobb	meal	0-40	35	1, 2, 3
8	Ahmed et al. (2015)	Egypt	Asia	480	4	Cobb	meal	0-20	42	1, 2 3
9	An et al. (2016)	Korea	Asia	600	5	Ross	meal	0-15	35	1, 2, 3
10	Gorski et al. (2017)	USA	NA	280	5	Leghorn	meal	0-40	37	1, 2, 3
11	Payvastegan et al. (2017)	Iran	Asia	180	4	Ross	meal	0-30	42	1, 2, 3
12	Radfar et al. (2017)	Canada	NA	120	3	Ross	meal	0-30	35	1, 2, 3
13	Disetlhe et al. (2018)	S. Africa	Africa	220	4	Ross	meal	0-17.5	42	1, 2, 3
14	Ghazalah et al. (2021)	Egypt	Asia	160	4	AA	meal	0-90	39	1, 2, 3
15	Elbaz (2021)	Egypt	Asia	320	4	Ross	meal	0-20	42	1, 2, 3
16	Olukomaiya et al. (2021)	Australia	Oceania	240	6	Ross	meal	0-30	21	1, 2, 3
17	Ajao et al. (2022)	USA	NA	540	6	Cobb	meal	0-10	32	1, 2, 3
18	Elbaz et al. (2023)	Egypt	Asia	500	5	Ross	meal	0-20	42	1, 2, 3
19	Hosseinpoor et al. (2023)	Iran	Asia	490	7	Ross	meal	0-15	49	1, 2, 3

NBT= Number of birds in the study; NT= number of treatments; 1= Feed intake; 2 =Average daily gain; 3= Feed conversion ratio. IL= Inclusion level; OF= offered form; SP= Study period indays; NA= North America; SA= South America; AA= Arbor Acres

The SMD was declared to be significant when zero was not included in the 95% CI. Heterogeneity (I^2^), which detects variability between studies was tested using Q-statistics and it describes the differences in SMD for each measured outcome (Higgins and Thompson, 2002). The degree of heterogeneity was categorized as none (0 < *I*^2^ ≤ 25%), low (25% < *I*^2^ ≤ 50%), moderate (50% < *I*^2^ ≤ 75%), and high (*I*^2^ > 75%) (Higgins et al. 2003) and was considered significant at 5%. The quantity of I^2^ accounted for by the moderators was determined through meta-regression analysis. The CM's effect on broilers’ growth traits was determined via a subgroup analysis. Sensitivity analysis was done using a standard method (Lean et al., 2009). In addition, publication bias that measures the failure of authors and journal editors to publish articles with insignificant results was evaluated via funnel plot methods. However, Jennions et al. (2013) revealed that the outcomes of a meta-analysis are deemed robust, notwithstanding the existence of publication bias when Nfs is higher than [5 × (n) + 10], where n = number of comparisons.

## Results

### Characteristics of articles used in the meta-analysis

**Fig 2:**
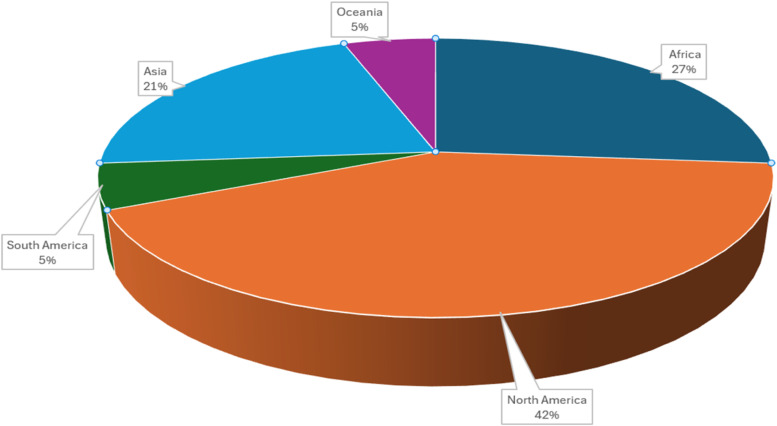
Pie chart showing proportion of articles included in the meta-analysis by continent.

A total of 100 publications were identified in various search engines of which 19 were eligible and selected for the meta-analysis (Table 1). The eligible articles showed that most of the studies were published between 2001 and 2023 (Table 2) from four continents (North and South America, Asia, and Africa) (Fig 2) in seven different countries (USA, Iran, Canada, Brazil, Egypt, South Africa, and Korea). A total of 6360 broilers from Ross, Cobb, heritage, leghorn, and Arbo acres strains aged between 1 to 49 days were used and CM was used to replace soybean meal (SBM) at inclusion levels ranging from 0 to 90%. Nevertheless, all 19 selected studies were evaluated for FI, ADG, and FCR.

**Table 2:** Number of publications in a year included in the meta-analysis.

Year of publications	Numbers of publications
2001	1
2008	1
2009	1
2011	1
2012	2
2014	1
2015	1
2017	1
2018	3
2021	1
2022	3
2023	3

### Feed intake

**Fig 3:**
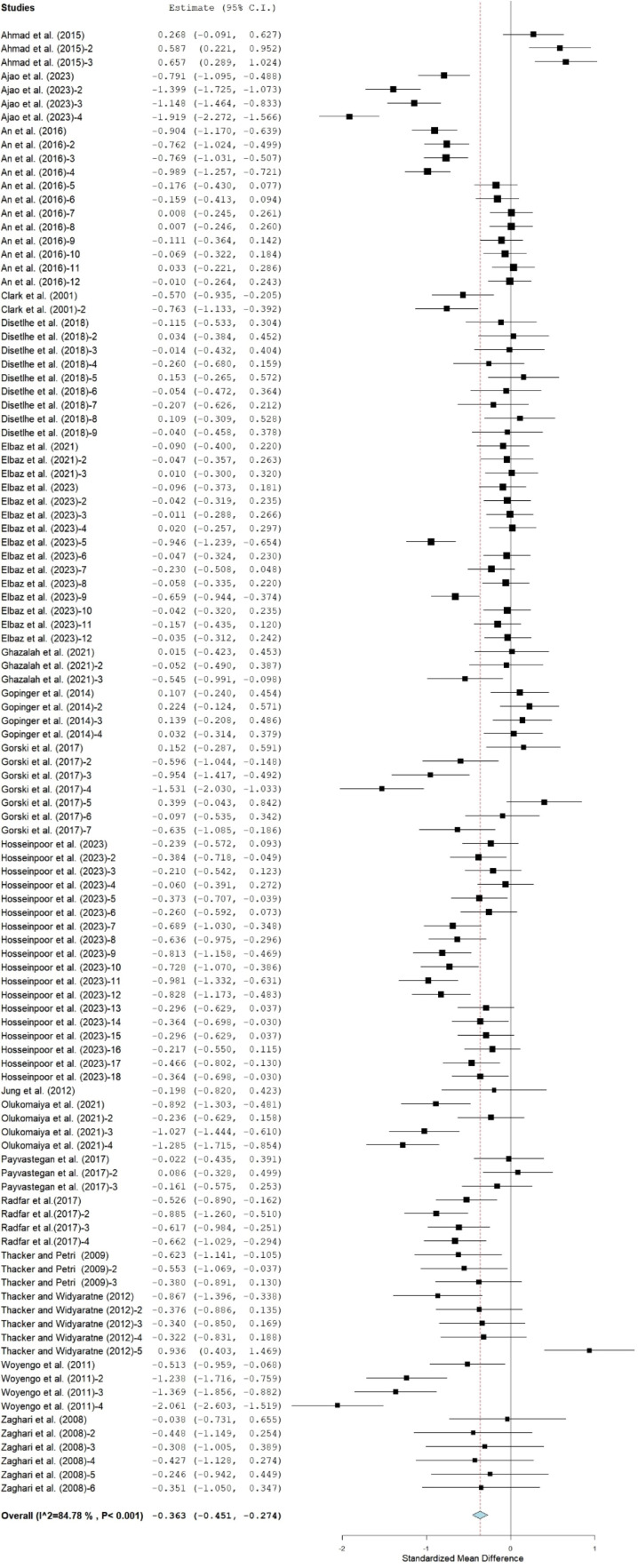
Forest plot of FI of broiler chickens fed canola meal. CI = confidence interval; FI= feed intake; I^2^ = Inconsistency index. The solid vertical line depicts a mean difference of zero (0) or no effect. Points to the left of the no effect line (zero) depict a decrease in FI and opposite depicts an increase in F1. Individual square in the plot represents the mean effect size for each experiment, while the upper and lower 95% CI for the effect size are the line that joined the squares. The dotted line with the diamond at the base showing the 95% CI depicts the pooled estimation. I^2^ = inconsistency index is a measure of variance above chance among articles utilized in the analysis. Pooled estimation is considered significant when the line of no effect did not touch the diamond at the bottom of the forest plot (Koricheva et al. 2013)

Pooled effects revealed that broiler chickens fed canola meal-based diets (**CMBD**) had significantly lower FI (SMD = −0.36; 95% CI = -0.45 to -0.27; Fig 3) than the control group. The subgroup analyses of the effect of moderators on FI are shown in Table 3. Cobb and Leghorn strains consuming treatment diets (CMBD) had similar FI to the control group, whereas lower (P<0.05) FI was observed in Ross and Arbor acres strains on treatment diets. Mixed gender broiler chickens reared on CMBD had comparable FI with those on the control diet. Conversely, male chickens and those whose gender was not stated had lower FI when offered CMBD compared to the control group. Broilers fed CM at 1-10% inclusion rates had comparable FI while higher inclusion rates (11- 40%) reduced FI compared to the control group. However, FI was consistently reduced throughout all the stages of development of the broilers when compared to the control group. In comparison to the controls, broilers fed untreated, enzyme and amino acids supplemented, fermented, and gamma irradiated CM had lower (P<0.05) FI, whereas those fed CM supplemented with humic acids or treated with a combination of fermentation and enzymes had comparable FI to those on control diets.

**Table 3:** Effects of canola meal on feed intake of broiler chickens

	Random Effects				Heterogeneity	
Subgroup	Nc	SMD	95%, CI	*p-value*	I^2^ (%)	*p-value*
Broiler strain						
Cobb	19	-0.01	-0.36, 0.17	0.48	89.2	0.00
Ross	72	-0.39	-0.48, -0.31	0.001	77.2	0.00
Arbor Acres	5	-0.40	-0.70, -0.10	0.009	62.7	0.03
Leghorn	6	-0.27	-0.70, 0.13	0.18	80.7	0.00
Gender						
Mixed	12	0.11	-0.07, 0.28	0.22	53.4	0.14
Male	62	-0.48	-0.62, -0.35	< 0.001	87.2	0.00
Not stated	33	0.31	-0.42, -0.21	< 0.001	71.7	0.00
Inclusion levels						
1-10	28	-0.33	-0.52, 0.15	0.28	87.0	0.00
11-20	61	-0.29	-0.38, -0.20	< 0.001	75.6	0.00
21-30	12	-0.60	-0.90, -0.29	< 0.001	81.5	0.00
31-40	2	-0.24	-0.90, -0.29	< 0.001	75.0	0,00
Stage of development						
Starter	43	-0.52	-0.647, -0.39	< 0.001	79.6	0.00
Finisher	16	-0.46	-0,65, -0.27	< 0.001	77.0	0.00
Overall	44	-0,12	-0.21, -0.03	0.01	7.0	0.00
Treatment methods						
Enzyme	21	-0.30	-050, -0.97	0.004	85.3	0.00
Amino acids	3	-1.10	-1.46, -0.76	< 0.001	72.6	0.03
Untreated	52	-0.35	-047, -0.24	< 0.001	79.5	0.00
Humic acids	3	-0.04	-0.28, 0.51	0.77	0.0	0.99
Fermentation	14	-0.33	- 0.49, -0.16	< 0.001	71.5	0.00
Ferm + Enzyme	3	-0.02	-0.18,0.14	0.77	0.0	0.92
Gamma radiation	6	-0.30	-0.59, -0.02	0.04	0.0	0.97

Ferm + Enzyme= fermentation and enzyme; Nc= number of comparisons; SMD = standardised mean differences; CI = confident interval; I^2^ = Inconsistency index.

### Average weight gain

**Fig 4:**
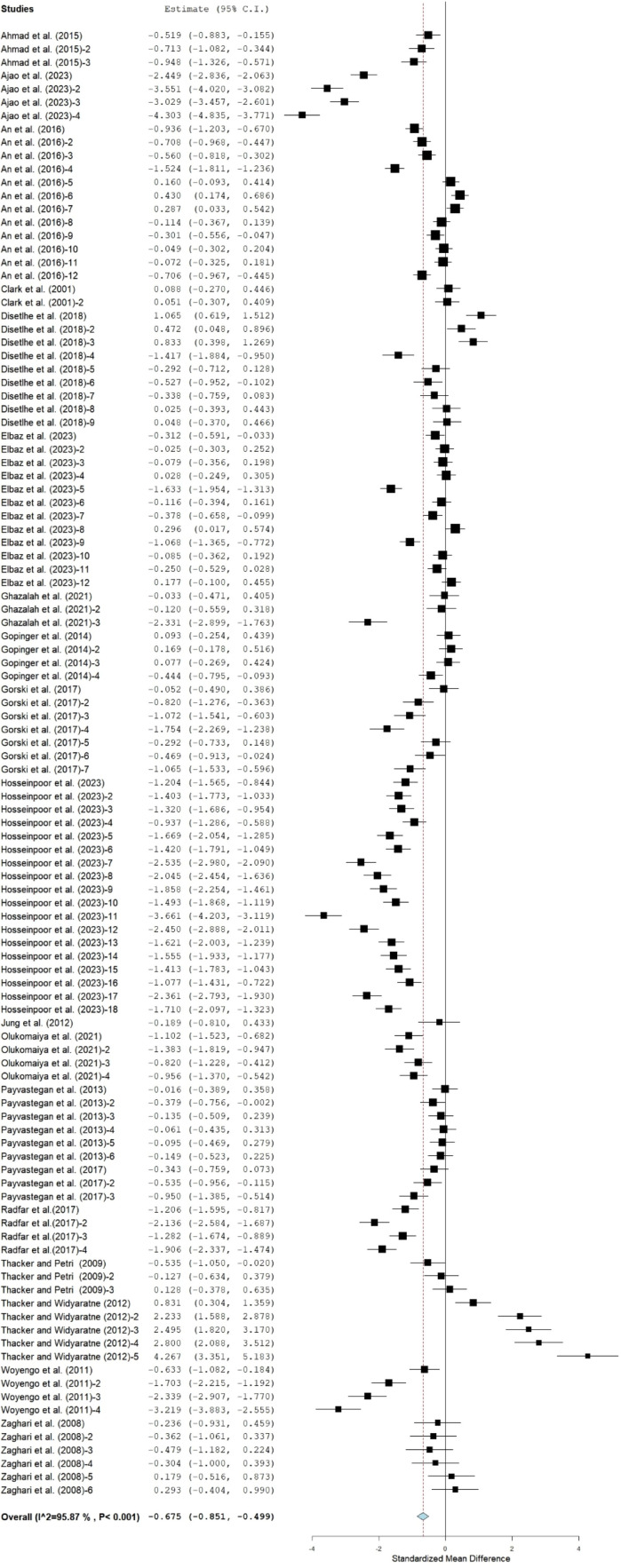
Forest plot of average daily gain (ADG) of broiler chickens fed canola meal. CI = confidence interval; I ^2^ = Inconsistency index. The solid vertical line depicts a mean difference of zero (0) or no effect. Points to the left of the no effect line (zero) depict a decrease in ADG. and opposite depicts an increase in ADG. Individual square in the plot represents the mean effect size for each experiment, while the upper and lower 95% CI for the effect size are the lines that joined the squares. The dotted line with the diamond at the base showing the 95% CI depicts the pooled estimation. I^2^ = inconsistency index is a measure of variance above chance among articles utilized in the analysis. Pooled estimation is considered significant when the line of no effect did not touch the diamond at the bottom of the forest plot (Koricheva et al. 2013).

As displayed in Fig 4, broilers fed on CMBD had significantly lower ADG (SMD = −0.68; 95% CI = -0.85 to -0.50) compared to the control group. Results of subgroup analyses of the effect of moderators on ADG that are presented in Table 4 showed that ADG was only similar between CMBD and control groups of Abor acres broiler chickens whereas for Cobb, Ross and Leghorn strains, CMBD birds had lower ADG compared to the control birds. Broiler chickens on CMBD, drawn from studies that used mixed and male gender broiler chickens, had lower ADG compared to those on the control. In contrast, studies that did not state the gender of broiler chickens reported comparable ADG between birds on CMBD and control diets. Broiler chickens that received diets containing CM at 1 – 40% recorded lower ADG compared to the control. Likewise, broiler chickens offered CMBD at starter and finisher stages of development had depressed ADG. Broilers fed on CMBD treated with humic acid and gamma irradiation had comparable ADG to those on control diets. In contrast, broilers fed CMBD supplemented with enzymes and amino acids, untreated, fermented, or treated with a combination of fermentation and enzymes exhibited significantly lower ADG compared to the control groups.

**Table 4:** Effects of canola meal on average daily gain of broiler chickens

	Random Effects				Heterogeneity	
Subgroup	Nc	SMD	95% CI	*p*-value	I^2^ (%)	*p*-value
Strain						
Cobb	20	-0.78	-0.35, -0.21	0.007	97.5	0.00
Ross	77	-0.66	-0.86, -0.46	<0.001	95.7	0.00
Arbor Acres	5	-0.44	-1.16, 0.28	0.22	93.2	0.00
Leghorn	7	-0.78	-1.19, -0.37	< 0.001	82.1	0.00
Gender						
Male	12	-0.20	-0.59, -0.20	0.33	90.7	0.00
Mixed	68	-0.54	-0.77, -0.30	<0.001	95.9	0.00
Not stated	30	-1.16	-1.47, 0.84	<0.001	96.3	0.00
Inclusion levels						
1-10	32	-0.43	-0.78, -0.08	0.02	96.7	0.00
11-20	62	-0.70	-0.92, -0.47	<0.001	95.7	0.00
21-30	12	-0.83	-1.20, -0.46	<0.001	87.0	0.00
31-40	4	-1.92	-3.13, -0.70	0.002	95.7	0.00
Stage of development						
Starter	48	-0.53	-0.82, -0.23	<0.001	96.1	0.00
Finisher	18	-1.01	-1.50, -0.52	<0.001	96.4	0.00
Overal1	44	-0.69	-0.93, -0.45	<0.001	98.3	0.00
Treatment methods						
Enzyme supplementation	21	-0.98	-1.28, -0.67	<0.001	92.9	0.00
Amino acids	4	-3.32	-4.07, -2.57	<0.001	91.1	0.00
Untreated	60	-0.37	-0.58, -0.16	<0.001	94.7	0.00
Humic acids	3	0.12	-1.65, 0.88	0.76	89.9	0.00
Fermentation	13	-1.40	-1.93, -0.87	<0.001	96.6	0.00
Fermentation + Enzymes	3	0.17	0.006, 0.33	0.04	0	0.41
Gamma radiation	6	-0.15	-0.44, 0.14	0.30	0	0.57

Nc= number of comparisons; SMD = standardised mean differences; CI = confident interval; I^2^ = Inconsistency index.

### Feed conversion ratio

**Fig 5:**
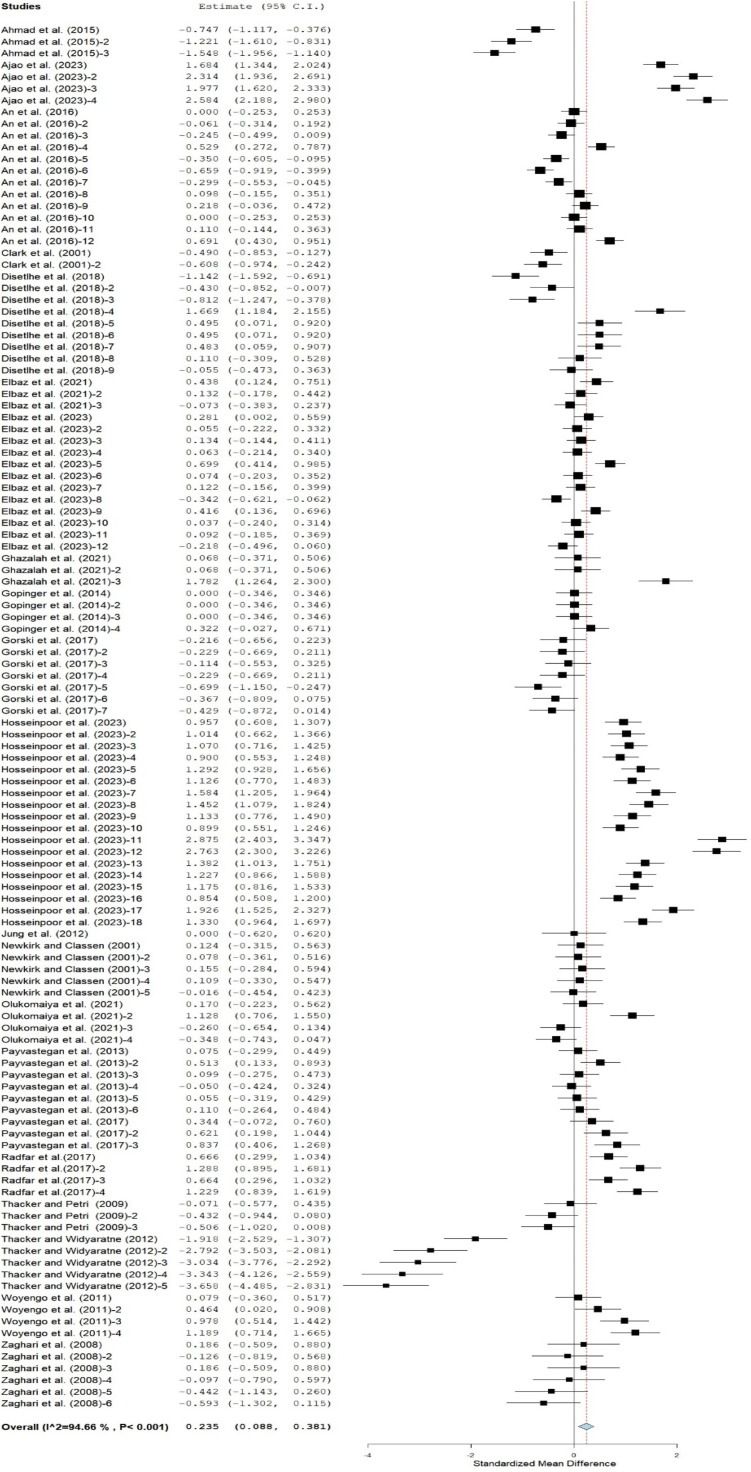
Forest plot of feed conversion ratio (FCR) of broiler chickens fed canola meal. CI = confidence interval; I ^2^ = Inconsistency index. The solid vertical line depicts a mean difference of zero (0) or no effect. Points to the left of the no effect line (zero) depict a decrease in ADG. and opposite depicts an increase in ADG. Individual square in the plot represents the mean effect size for each experiment, while the upper and lower 95% CI for the effect size are the lines that joined the squares. The dotted line with the diamond at the base showing the 95% CI depicts the pooled estimation. I^2^ = inconsistency index is a measure of variance above chance among articles utilized in the analysis. Pooled estimation is considered significant when the line of no effect did not touch the diamond at the bottom of the forest plot (Koricheva et al. 2013).

Pooled results revealed a significantly poor FCR (SMD = 0.24; 95% CI = 0.09 to 0.38) in broilers fed CMBD compared to birds on the control diet (Fig 5). Table 5 shows the effects of CMBD on the FCR of broilers. In this table, Cobb, Abor acres, and Hubbard strains were not significantly affected by CM inclusions. On the other hand, Ross birds on CMBD showed a significantly poorer FCR compared to those in the control group. In contrast, the leghorn birds on CMBD had the best FCR compared to the control group. Broiler chickens on CMBD, drawn from studies that used mixed and male gender chickens, had similar FCR to the control group. In contrast, studies that did not state the gender of broiler chickens reported better FCR for birds on CMBD compared to the control group. Broilers fed CM at 1 – 10, 11 – 20, and 21 – 30% inclusion levels had a comparable FCR to the control group, however, broilers fed diets containing CM at higher levels (31 – 40%) had poorer FCR compared to the control group. Poor FCR values were evident at both the starter and finisher phases, as well as throughout the entire feeding period. Subgroup results indicated that enzyme-treated, amino acid-supplemented, untreated, and fermented CM increased the FCR of broilers. However, birds offered CM treated with humic acids, toasting, gamma irradiation and mixtures of fermentation and enzymes had similar FCR as the control group.

**Table 5:** Effects of canola meal on feed conversion ratio of broiler chickens.

	Random Effects				Heterogeneity	
Subgroup	Nc	SMD	95% CI	p-value	I^2^ (%)	p-value
Strain						
Cobb	20	0.31	-0.21, 0.83	0.24	97.2	0.00
Ross	72	0.50	0.35, 0.64	< 0.001	92.7	0.00
Arbor Acres	5	0.15	-0.59, 0.89	0.69	93.7	0.00
Leghorn	7	-0.32	-0.49, -0.16	< 0.001	0.0	0.63
Hubbard	5	0.90	-0.11, 0.29	0.10	0.0	0.99
Gender						
Mixed	18	-1.04	-0.45, 0.25	0.56	92.1	0.00
Male	68	0.04	-0.15, 0.24	0.67	94.3	0.00
Not stated	32	0.81	0.57, 1.06	< 0.001	94.6	0.00
Inclusion levels						
1-10	29	0.17	-0.32,0.47	0.27	95.5	0.00
11-20	64	0.49	-0.32, 0.67	0.07	93.5	0.00
21-30	13	0.13	-0.16, 0.42	0.37	81.9	0.00
31-40	4	0.76	0.67, 1.57	< 0.001	92.8	0.00
Stage of development						
Starter	45	0.30	0.10, 0.50	0.002	92.1	0.00
Finisher	22	0.53	0.16, 0.10	0.005	95.9	0.00
Overall performance	43	0.37	0.15, 0.59	0.001	94.2	0.00
Treatment methods						
Enzyme	21	0.35	0.001, 0.70	0.05	94.5	0.00
Amino acids	4	2.13	1.75, 2.52	<0.001	77.3	0.004
Untreated	53	0.21	0.06, 0.35	0.005	89.0	0.00
Humic acids	3	-0.12	-0.86, 0.61	0.74	88.8	0.00
Fermentation	14	1.09	0.64, 1.53	<0.001	95.6	0.00
Fermentaion+Enzymes	3	-0.17	-0.40, 0.70	0.17	53.3	0.11
Toasting	5	0.90	-0.11, 0.29	0.37	0.0	0.99
Gamma radiation	6	0.09	-0.43, 0.14	0.37	0.0	0.56

Nc= number of comparisons; SMD = standardised mean differences; CI = confident interval; I^2^ = Inconsistency index.

### Meta-analysis and bias analysis

**Fig 6:**
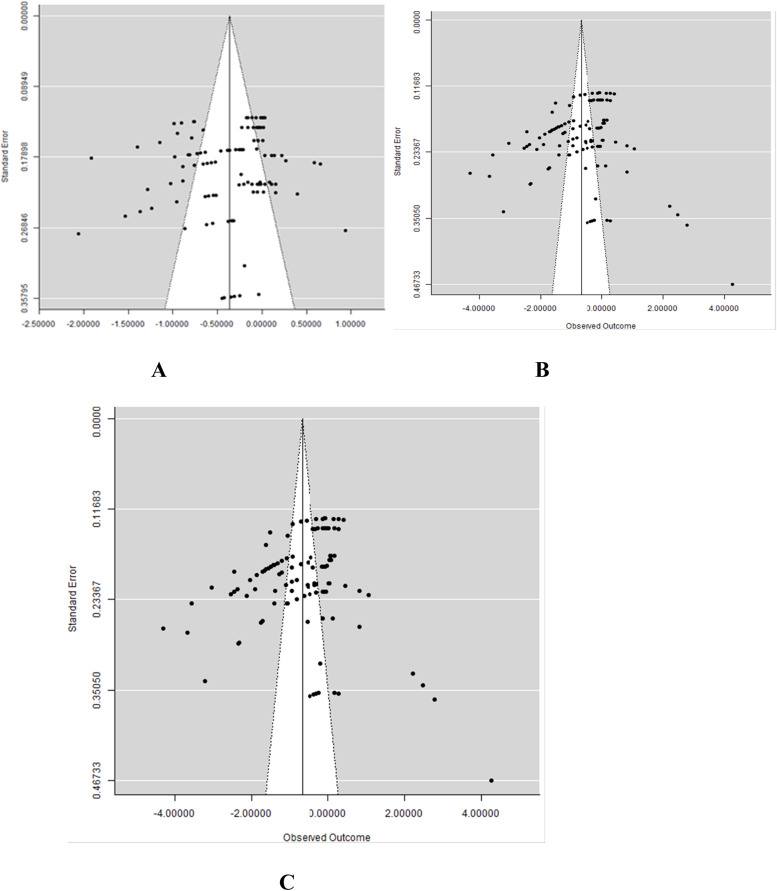
Funnel plots of the effects of canola meal-based diets on feed intake (A), average daily gain (B), and feed conversion ratio (C) in broilers.

Meta-regression analysis on the influence of moderators on FI, ADG, and FCR of broiler chickens fed CMBD showed that strain and inclusion levels had no significant effect on FI, ADG, and FCR (Table 6). However, the stage of development was associated with FI (*P* <0.001*;* R^2^ = 24.69%), but not with ADG and FCR in this meta-regression analysis. Likewise, gender and CM treatment methods had strong association with FI, ADG, FCR. The results of publication bias analysis are presented in Fig 6 and reflect a strong tendency for medium studies to be associated with greater positive effects for FI, ADG, and FCR. The funnel graphs (Fig 6) were asymmetrical indicating the presence of publication bias among the 19 articles. Rosenberg's fail-safe number (Nfs) was further computed for evidence of publication bias. The Nfs for the database for FI, ADG, and FCR are 15402, 49604, and 10377, which were 146.69, 551.16, and -98-fold higher than the thresholds of 105 (5 × 19 + 10), 90 (5 × 16 + 10) and 105 (5 × 19 + 10), respectively, required for the mean effects size to be declared robust.

**Table 6:** Meta-regression comparing the associations between covariates and growth parameters.

Outcomes	Covariates	Intercept	Q_M_	Estimate	*df*	*p-*value	R^2^ (%)
Feed intake	Strain	-0.10	8.04	0.13	4	0.09	4.03
	Gender	0.10	16.32	-0.17	2	<0.001	14.51
	Inclusion levels	-0.34	5.09	0.13	3	0.17	2.12
	Stage of development	-0.12	26.70	0.10	2	<0.001	24.69
	Treatment methods	-0.30	17.15	0.12	7	0.02	12.46
ADG	Strain	-0.78	0.56	1.42	4	0.97	0.00
	Gender	-0.19	8.49	1.29	2	0.001	5.90
	Inclusion levels	-0.43	6.00	3.00	3	0.11	2.71
	Stage of development	-0.70	2.25	1.37	2	0.33	0.09
	Treatment methods	-0.98	47.66	0.96	6	<0.001	29.73
FCR	Strain	0.31	8.47	0.59	5	0.13	3.36
	Gender	-0.10	17.8	0.84	2	<0.001	12.77
	Inclusion levels	0.17	5.48	0.60	3	0.14	2.33
	Stage of development	0.37	1.15	0.62	2	0.56	0.00
	Treatment methods	0.35	53.9	0.42	8	<0.001	31.64

ADG = Average daily gain; FCR = feed conversion ratio; Q_m_ = coefficient of moderators; df = degree of freedom; R^2^ = amount of heterogeneity accounted for by covariate

## Discussion

### Growth performance

In this meta-analysis, the inclusion of CM in broiler diets reduced FI, ADG, and feed utilization efficiency, which could be due to higher amounts of fiber, non-starch polysaccharides, and glucosinolates found in CM. Indeed, these anti-nutritional factors (ANFs) have been reported to suppress growth performances of broilers (Sabbahi et al., 2023). Feeding poultry with a fiber (non-starch polysaccharides)-rich diet increases mucin secretion from goblet cells (Duangnumsawang et al., 2021), which slows the diffusion of endogenous digestive enzymes and their substrates at the mucosal surface, thereby reducing the digestion and absorption of nutrients (Leala et al., 2017). In addition, the regulation of feed intake and weight gain (melanocortin systems) in the hypothalamus is controlled by thyroid hormone (Cicatiello et al., 2018), which is suppressed by glucosinolates and its co-products. The observed significant reduction in FI agrees with the findings of Mnisi and Mlambo (2018b) who found that 17.5% CM reduced FI in 10-week-old Japanese quail. It was expected that including CM in broiler diets would enhance growth performance, as modern canola varieties have been bred with lower concentrations of glucosinolates. However, the reduced growth performances in broilers fed test diets when compared with the control group indicate that plant breeding may not have eliminated all glucosinolates. Indeed, CM is known to be bitter when used at higher inclusion levels (Mailer et al., 2008). This could explain the poor performance of broilers at higher CM inclusion levels as observed in this meta-analysis. Therefore, the observed results are coherent and indicate that the high inclusion of CM in broiler diets suppresses growth performance while low inclusion rates consistently improved performance or at least resulted in similar performance metrics as the control.

### Explanatory moderator variables

#### Broiler strains

This meta-analysis indicates that relationships exist between strain and FI in broilers fed CMBD. The comparable FI between CMBD and control groups in the Cobb 500 and Leghorn strains indicate the ability of these strains to overcome the negative effects of ANFs such as glucosinolate, erucic acid, sinapine, and tannins. Conversely, the reduced FI in the Ross and Arbor acres strains on CMBD implies that these strains have lower tolerance for ANFs than Cobb 500 and Leghorn. Similarly, genotypic differences in FI of chickens have been previously demonstrated by Sebola et al. (2015) in indigenous chickens and Ogbuewu et al. (2024) in broiler chickens. The comparable FI in Cobb 500 and Leghorn strains indicates that the digestive system of these strains has the capacity to utilise CM-containing diets. Similar ADG between CMBD and control groups in Abor acres indicates the ability of this strain to utilize the protein in CM. In contrast, Cobb 500, Ross 308, and Leghorn birds on CMBD had poor ADG implying inability of these strains to extract, break, assimilate, and utilize protein in CM, possibly due to the presence of fiber. Indeed, high fiber is known to negatively affect the process of digestion, assimilation, and utilization of nutrients (Adams et al., 2018). There are limited published studies on the effect of dietary CM on growth performance of different broiler strains, making comparisons with the results of the present study challenging; thus, further research is needed. Furthermore, lower ADG could be associated with low FI in this study. Nevertheless, this meta-analysis revealed Abor acres as the strain that is better able to overcome ANFs effects in CMBD.

At the farm level, higher FCR indicates higher feed requirements per unit of production output, which is influenced by the quality of feed and management practices. In this meta-analysis, a possible explanation for poor FCR in Ross 308 could be the negative effects of fiber on nutrient digestibility and absorption. Contrary to this finding, An et al. (2016) found that increasing CM inclusion levels did not affect FCR in Ross 308. The disparity could be due to differences in the gender of birds used in the studies. In contrast, the FCR in Cobb 500, Abor acres, and Hubbard chickens on CMBD did not significantly differ from control birds implying that these strains are better able to utilize nutrients in CM. Indeed, Gopinger et al. (2014) confirmed that Cobb 500 birds on CMBD had the same FCR as those fed control diet. Conversely, Leghorn was reported to have low FCR in this study indicating a higher capacity to utilize fibrous diets and tolerate anti-nutritional compounds such as glucosinolates, a results similar to that of Manyeula et al., 2020 in Potchefstroom Koekoek chickens. Accordingly, it can be deduced that the potential to digest and utilize CMBD depends on bird strain. Based on the present findings, it can be concluded that Abor acres are more suitable for CMBD.

#### Gender of birds

The explanatory moderator analysis in this study showed that gender influences growth performance of broiler chickens. Lower FI in CMBD-fed birds compared to control groups was observed in males and chickens whose gender was not stated. A similar pattern has been reported in broiler chickens fed rapeseed meal (Manyeula et al., 2025). Mixed gender birds on CMBD had similar FI, while birds whose gender was not stated had similar ADG, as control groups, suggesting that CM glucosinolates and its co-products intake were within the acceptable threshold for these groups. The lower ADG in mixed gender and male broiler chickens on CMBD could be due to perosis, liver haemorrhage, and enlargement of thyroid and liver caused by glucosinolates (Raeesi et al., 2012; Taraz et al., 2006). These findings are in harmony with the results of Disetlhe et al. (2018), who reported a decline in weight gain of mixed gender broiler chickens when fed diets containing 17.5% CM. In contrast, Mnisi and Mlambo reported similar ADG in a mixed gender Japanese quail fed diets containing 17.5% CM. The observed disparity might be attributed to differences in the experimental animals used (broilers vs quail). However, gender had no influence on FCR of the birds

### Inclusion levels

The inclusion level of CM in broiler diets was identified as a limiting factor in this meta-analysis as it significantly affected FI. Normally broilers consume feed to fill their gut if not limited by dietary toxicities, environment, management practices, or disease factors (Ferket and Gernat, 2006). In the current study, dietary inclusion of CM at <10% promoted comparable FI as control diets, indicating that low inclusion levels can be used to replace SBM without impacting FI negatively. This observation agrees with the findings of Manyeula et al. (2018) in indigenous chickens, Disetlhe et al. (2018) in broiler chickens, and Mnisi and Mlambo in Japanese quail fed CMBD. The reduction in FI of broilers fed CM at >11% inclusion rates indicates that higher inclusion rates alter the physicochemical properties of diets, thus negatively affecting their palatability and functional properties (Manyeula et al., 2018). Similarly, Woyengo et al. (2011) observed that dietary inclusion of CM at incremental levels of 0 to 40% linearly decreased FI in broiler chickens. Weight gain is one of the key metrics for assessing the effectiveness of sustainable broiler production as it is influenced by FI (Tarsani et al., 2019). In this meta-analysis, reduced ADG in diets containing CM at all inclusion levels is linked with an imbalance in the amino acid composition of the diets (Rehman et al., 2019). Amino acids are responsible for muscle development and productivity (Soglia et al., 2021) and when essential amino acids are present in adequate amounts, birds can utilize all other amino acids in feed (Hung et al., 2020). Indeed, NRC (2012) reported lower lysine content in CM (5.52%) than in SBM (6.20%). The poor ADG in broilers fed CMBD at all inclusion levels is corroborated by Moraes et al., (2018), who reported reduced weight gain with increased inclusion levels of CM in broiler diets. This poor ADG at all inclusion levels could be due to high fiber contents (Newkirk et al., 2003) that increase the rate of passage of the digesta, thus, leading to less digestibility hence poor ADG. In this meta-analysis, inclusion levels were another significant predictor of FCR. However, subgroup analysis showed that inclusion levels at 1 – 30% had comparable FCR with control groups suggesting that, at these levels, broiler chickens can extract nutrients from these diets despite the presence of glucosinolates in CM. Moraes et al. (2018) reported a similar pattern in broilers fed diets containing 0 – 30% CM. On the other hand, inclusion levels beyond 30% had poor FCR indicating the inability of the broilers to extract nutrients from diets with high levels of CM. Similarly, Ghazalah et al. (2021) reported poor FCR in broilers fed a diet containing 90% CM. This suggests that the performance of broilers fed CM in place of SBM is influenced by inclusion levels. Accordingly, when formulating diets containing CM for broilers, the inclusion level should range between 10 and 30%.

### Stage of development

Results indicate that the stage of development is a significant predictor of FI, ADG, and FCR. In this study, broilers fed CMBD had significantly poor FI, ADG, and feed utilization efficiency in comparison with the control birds at both the starter and grower phases. This indicates that CMBD compromise growth performance at all stages of the broiler's development due to high fiber and glucosinolates contents. This finding suggests that the level of CM glucosinolates, which impart a bitter taste, is above the tolerance limit for broilers. According to Egorova and Lenkova (2015), glucosinolates are highly water soluble and remain functional even after oil extraction and conversion of the seed to a meal. In a related study, Ahmad et al. (2007) observed that the effects of glucosinolate is mostly pronounced in young birds than in older ones. The observed inferior performance at all stages of development contradicts the findings in Japanese quail and starter and finisher broiler chickens (Aljubori et al., 2017; An et al., 2016) where CM did not influence FI. Like the results of this current study, Naseem et al. (2006) found reduced FI in broilers fed diets containing 25% CM over a 28-day feeding period.

### Treatment methods

Numerous treatment methods have been used to ameliorate the effects of fiber and glucosinolates when CM has been used in diets of monogastrics to enhance its nutritive quality (Mnisi et al., 2017; Mnisi and Mlambo, 2018a; 2018b). This study showed that broilers fed diets containing CM treated with humic acid and a combination of fermentation and enzyme treatment had comparable FI to those fed control diets, indicating the ability of these two methods to counteract the anti-nutritional effects of glucosinolates and fiber (Heyer et al., 2021). This finding agrees with Olamiti et al. (2020), who reported that the addition of exogenous enzymes and malting degrades the complicated structures of protein and carbohydrates into simple and soluble components in grains. Contrary to our expectations, this meta-analysis showed that other treatment methods (i.e., supplementation of enzymes and amino acids and gamma radiation) resulted in decreased FI, indicating the inability of these strategies to ameliorate the negative effects of glucosinolates and fiber. In view of this, it can be deduced that various treatment methods counteract ANFs differently (Samtiya et al., 2020) in CM. It can be concluded that humic acid and a combination of fermentation and exogenous enzymes are the best strategy to deal with ANFs present in CM.

In this study, it was observed that weight gain in broilers fed diets containing canola treated with humic acid and gamma radiation did not differ from those fed controls suggesting that these methods are effective in dealing with fiber and secondary metabolites in CM. Indeed, Toghyani et al. (2010) reported that growth-promoting agents such as humic acid have been shown to improve the digestion and utilization dynamics of some protein source ingredients. Indeed, humic acid increases the counts of beneficial gut bacteria (Schepetkin et al., 2003) as well as villi length and crypt depth leading to improved nutrient utilization (Chaveerach et al., 2004) due to increase in absorption area (Arif et al., 2019). On the other hand, the fermentation and enzyme cocktail treatment of CMBD improved ADG, demonstrating the method's potential to enhance metabolism and nutrient absorption. This improvement is facilitated by the breakdown of anti-nutritional factors (ANFs) such as phytic acid and fiber, and the promotion of digestive enzyme activity (Hamdi et al., 2018), resulting in increased free amino acids in the diet and more efficient protein hydrolysis (Escobar-Alvarez et al., 2022). These findings agree with an earlier study in which feeding diets containing fermented CM treated with exogenous enzymes to broiler chickens improved ADG (Elbaz et al., 2023). Feed utilization efficiency was significantly poor in broilers fed with enzyme-treated, amino acids-supplemented, untreated, and fermented CM, implying that these methods were not effective. In contrast, broiler chickens fed diets containing humic acid-treated, fermented, and enzyme-supplemented, toasted, and gamma-irradiated CM showed comparable FCR in this study. This suggests that these treatments improved the nutritional quality of CM. These findings support the need for treatment strategies to improve the nutritive value and feed quality of CM. This finding is consistent with those in published studies showing that treatment methods improve feed quality of some protein source ingredients (Elbaz et al., 2023).

### Meta-regression

The results of the meta-regression showed that the stage of development has a significant correlation with FI in broiler chickens on CMBD. This finding is in line with Yang et al. (2020), who reported a plateau after 14 days of age when determining the relationship between age and metabolizable and digestible energy in broiler chickens. Likewise, there is a significant association between covariates (gender and treatment methods) and FI, ADG, and FCR indicating that the effects of dietary CM on these three parameters in broiler chickens were explained by gender and treatment methods, in agreement with reports by Niu et al. (2023a, b). Additionally, Yuan et al. (2024) and Manyeula et al. (2025) observed an association between growth parameters and gender in Xue Shan and broiler chickens, respectively.

### Limitations and strengths of the meta‑analysis

The study assessed the effects of CMBD on FI, ADG, and FCR in broiler chickens. Studies included in the meta-analysis may have employed different approaches to measure parameters of interest, which could be a limitation. In addition, glucosinolates levels were not measured or standardized across the studies used in this meta-analysis, which could have contributed to observed variation in reported outcomes. On the other hand, this meta-analysis summarises all published evidence on the effects of feeding broiler chickens a diet containing CM on FI, ADG, and FCR, thus it establishes the standards for measuring and reporting the soybean replacement value of CM in broiler chickens in the future.

## Conclusions and future research direction

The results of this meta-analysis revealed the negative impact of incorporating CM in broiler diets on growth performance. The pooled results showed that broilers fed CMBD had poor FI, ADG, and FCR, which were directly influenced by the studied covariates (strain, stage of development, inclusion level, and treatment methods). Meta-regression showed that treatment methods were predictors of FI, ADG, and FCR while the stage of development was a predictor for FI only. However, humic acid could be recommended as a treatment to improve CM utilization and CM inclusion levels between 10 and 30% could be used to replace SBM in broiler diets. The meta-analysis provides new insights into the negative effects of CM in broiler chicken diets, which will guide farmers and poultry nutritionists intend on using CM in broiler diets. Further studies should be directed at effective CM treatment methods to improve its utilization as well as the optimal inclusion levels that will not hinder the growth performance of broiler chickens.

## Declaration of competing interest

The authors declare that they have no known competing interest that could have appeared to influence the work reported in the present study.
